# Optimising Bait for Pitfall Trapping of Amazonian Dung Beetles (Coleoptera: Scarabaeinae)

**DOI:** 10.1371/journal.pone.0073147

**Published:** 2013-08-30

**Authors:** Charles J. Marsh, Julio Louzada, Wallace Beiroz, Robert M. Ewers

**Affiliations:** 1 Department of Life Sciences, Imperial College London at Silwood Park, Ascot, Berkshire, United Kingdom; 2 Departamento de Biologia, Universidade Federal de Lavras, Lavras, Minas Gerais, Brazil; University of Lancaster, United Kingdom

## Abstract

The accurate sampling of communities is vital to any investigation of ecological processes and biodiversity. Dung beetles have emerged as a widely used focal taxon in environmental studies and can be sampled quickly and inexpensively using baited pitfalls. Although there is now a wealth of available data on dung beetle communities from around the world, there is a lack of standardisation between sampling protocols for accurately sampling dung beetle communities. In particular, bait choice is often led by the idiosyncrasies of the researcher, logistic problems and the dung sources available, which leads to difficulties for inter-study comparisons. In general, human dung is the preferred choice, however, it is often in short supply, which can severely limit sampling effort. By contrast, pigs may produce up to 20 times the volume. We tested the ability of human and pig dung to attract a primary forest dung beetle assemblage, as well as three mixes of the two baits in different proportions. Analyses focussed on the comparability of sampling with pig or human-pig dung mixes with studies that have sampled using human dung. There were no significant differences between richness and abundance sampled by each bait. The assemblages sampled were remarkably consistent across baits, and ordination analyses showed that the assemblages sampled by mixed dung baits were not significantly different from that captured by pure human dung, with the assemblages sampled by 10% and 90% pig mixes structurally most similar to assemblages sampled by human dung. We suggest that a 10:90 human:pig ratio, or similar, is an ideal compromise between sampling efficiency, inter-study comparability and the availability of large quantities of bait for sampling Amazonian dung beetles. Assessing the comparability of assemblage samples collected using different baits represents an important step to facilitating large-scale meta-analyses of dung beetle assemblages collected using non-standard methodology.

## Introduction

Quantifying ecological processes and the effects of anthropogenic disturbance requires us to have an accurate and comparable representation of ecological assemblages. Inaccurate sampling may lead to spurious conclusions regarding the responses of species to anthropogenic processes and so it is essential that researchers implement standardised sampling protocols on appropriate indicator taxa.

Dung beetles are emerging as an increasingly popular focal taxon for ecological research [1–4]. They are cost-effective to survey [4], can be rapidly sampled using baited pitfall traps [5], and are sensitive to anthropogenic disturbances and habitat change [6]. They also fulfil several important functional roles including secondary seed dispersal, soil turbation, parasite suppression and nutrient cycling [7–9], and these functional roles can be easily manipulated in the field [10,11]. Furthermore, they may be separated into ecologically meaningful functional groups based upon diel activity, body size and one of three breeding strategies, rollers, tunnellers and dwellers [12,13], that determine rates of dung removal, seed dispersal and germination [8,14].

Dung beetles may be easily sampled using inexpensive baited pitfall traps [15]. However, the choice of bait has been largely driven by the idiosyncrasies of individual researchers rather than based upon empirical evidence and this can severely hinder the validity of inter-study comparisons [16]. By contrast, a large number of studies have investigated the resource preferences of dung beetle species [17–27]. Although some species are highly specialised, the majority of dung beetles have wide diet breadths and may be attracted to a variety of alternative baits, including dung, carrion and rotting fruit and fungi [28]. Dung-feeding species in particular seem remarkably generalised in diet and can be trapped with many dung types [28], although the type and size of dung used can have a significant effect on the number of species and individuals captured [18,19,24,29–33].

There has been little effort to quantify the abilities of different baits to successfully sample an assemblage and the question remains as to what is the optimal bait choice for ecological research. In general, omnivore dung captures a wider breadth of species than herbivore dung and carrion. Human dung, in particular, seems to sample a greater number of species and biomass than other baits (eg [24,28,29]). For example, Larsen et al. [28] found that all species except one attracted to non-human dung were also sampled by human dung, while collecting five times more individuals and twice the number of species than cow dung. Furthermore, human dung is available across the world wherever the researcher travels. Consequently, it has become the bait of choice in the majority of studies investigating the effects of habitat disturbance, particularly in the neotropics, although cattle and pig dung is also used in Africa and Asia [34,35]. However, a researcher can only provide fresh dung for around eight to ten traps per day, based on a standard bait size of 20g (personal observation). Unless they have particularly cooperative companions this can provide a major limiting factor on potential sampling effort.

A domesticated pig, conversely, is likely to produce over 20 times the weight of manure [36], and although not all will be useable as bait, it may nonetheless vastly increase the number of traps a researcher can set. As another omnivore, pig dung may also attract a wide range of species, although to our knowledge the richness of dung beetle assemblages sampled using pig dung has not been compared against human dung. Furthermore, pigs from rural households are largely fed household waste, and so likely has a diet almost equivalent to that of a human. By contrast, industrial pigs may have a very different diet and so would not be preferable. Finally, wild suiformes (pigs and peccaries) are found across the globe and so are natural sources of dung for many forest species.

If dung beetles do show preferences for specific types of dung, then potentially a mix of two or more dung types may catch a fuller complement of the assemblage and therefore provide a more complete inventory of the dung beetles present than a single dung type. Bait mixes (as opposed to trap arrays that consist of adjacent but separate pitfalls with different baits) are rarely used (eg [37]) and it remains untested if they capture a wider breadth of species. Here, we examine the efficacy of pig dung and pig-human dung mixes at sampling dung beetle assemblages compared to sampling using pure human dung. Given that human baited pitfalls have been implemented in the majority of previous studies, we specifically compare the assemblages sampled by human dung with those sampled by the mixes and pig dung. A successful bait will sample both a high number of species and individuals, but also an assemblage that is comparable to that collected by human dung to allow for valid comparisons with the previous literature.

## Methods

The study was carried out in the Jari region in the north-east Amazon, Pará, Brazil. Jari consists of ~65,000 ha of *Eucalyptus* plantations, ~45,000 ha of regenerating secondary forest and ~1.5 Mha of largely undisturbed primary forest. The study site was an area of largely undisturbed primary forest, large enough that sites could be considered independent of neighbouring *Eucalyptus* plantations (mean distance of sampling points from Eucalyptus = 763m, range = 443-1057m). Grupo Orsa kindly provided permission to carry out sampling at the study site and collecting permits were issued by the Ministério do Meio Ambiente (MMA-IBAMA).

### 2.1 Dung beetle sampling

We established 25 irregularly spaced sampling points separated by ~100m in order to achieve trap independence (mean distance to nearest neighbour = 96.8m; range = 83.8-122.2m [15]). A baited pitfall trap (20cm width, 15cm depth) was placed at each sampling point, buried flush with the ground and part-filled with a killing solution comprising of water, salt and detergent.

We investigated five bait combinations: a) pure human dung, b) a 10:90 pig-human mix, c) 50:50 pig-human mix, d) 90:10 pig-human mix, and e) pure pig dung. Hereafter we refer to bait mixes only by the percentage of pig dung (10%, 50% and 90%). The bait mixes were homogenised, separated into 20g gauze parcels and frozen until use. The number of species and individuals may be positively correlated with bait quantity [30], but 20g was chosen as this quantity is commonly used in dung beetle sampling (eg [38]). For each trap, bait was suspended beneath a lid directly above the pitfall. The lid acted as a rain cover as well as limiting the ability of beetles to directly access the bait. Bait type was assigned randomly to each point so that there were five replicates of each bait type. When using pig dung, the same precautions should be used as when using human dung as dung from domesticated pigs may contain transferrable pathogens.

Sampling was carried out over eight days in the late wet season between 14–22 July 2010. Traps 1-10 were set on the first day and the remaining traps set on the second day, and all traps operated over seven trap nights. Trap contents were collected after two, five and seven days, and bait replaced after two and five days. All individuals collected were oven dried and then identified to species level using a reference collection held at the *Universidade Federal de Lavras* (UFLA), where all samples were deposited. Species were identified using classifications developed by Vaz-de-Mello and Gardner (unpublished).

### 2.2 Analyses

The attractiveness of traps may be confounded by their location and the order in which they were sampled. First, traps that were set on the first sampling day may capture more individuals than traps set on the second due to a potential depletion effect [24]. Second, although traps were set ~100m apart, if beetles are attracted to dung over distances greater than 50m, then traps at the edge of the sampling array may capture more individuals than interior traps as they will have fewer competing near neighbours and therefore sample from a wider neighbourhood. We investigated if there were significant differences in the number of species sampled between edge and interior traps, and between traps set on the first and second sampling days through Welch’s t-tests.

Further analyses focussed on the ability of pig dung and human-pig dung mixes to sample the dung beetle assemblage and the similarity of these assemblages to those sampled by human dung.

#### 2.2.1 Sampling efficiency

We investigated two aspects of a baits ability to efficiently and accurately sample the assemblage. First, we investigated the proportion of the assemblage sampled by each bait. A successful sampling strategy should rapidly capture an almost complete representation of the assemblage. We calculated the mean and total number of species and number of individuals and the mean biomass sampled by each bait, and also visually compared trap-based accumulation curves to examine the rate at which species were sampled. Dry weight for each species was determined to 0.0001g as the mean of 15 individuals collected at the study site between 2009 and 2012. Where <15 individuals were available weights were cross-checked with available sources. Significance of differences between baits for mean richness, abundance and biomass were tested using Mann-Whitney tests. Second, as studies frequently remove species of low abundance prior to analyses, and that these species may also have large influences on richness estimates, we investigated the influence of rare and occasional species on estimates of species richness [39]. We determined the number of species in each bait type while sequentially removing singletons, doubletons and so on in order to ascertain their influence on diversity estimates.

#### 2.2.2 Inter-study comparability

We compared the composition of the assemblages sampled by the pig-based baits with pure human dung bait. As a large proportion of existing surveys have used human dung baits, it is important that any dung mixes sample an equivalent subset of the assemblage to allow for inter-study comparisons. First, we investigated the structure of rank abundance curves for each bait type, maintaining the rank order of species as observed in the traps baited with human dung. If a bait samples a very different assemblage from the assemblage sampled by human dung then the abundance plot will become deconstructed, with changes in the identity of the most abundant species. We also investigated the number of shared species between the pig-based bait assemblages and the human bait assemblage using Jaccard dissimilarity, and investigated if mean dissimilarities were higher between traps with different baits compared to between traps with the same bait. Furthermore, we grouped genera into functional groups representing different breeding strategies, body size and patterns of diel activity using pre-existing literature [40], and investigated if there were clear differences in the range and structure of functional groups collected by each dung type. We assigned the diel activity for a species within a given genera as the diel activity of the majority of species within that genera. There may be small intra-genera differences in diel activity that are obscured by assigning this functional trait at the level of genus rather than species, however, we lacked species-specific data for all species present at the study site.

Finally, we compared the assemblages sampled by each bait type using partial canonical ordination (RDA [41]). We investigated differences in assemblage composition using presence-absence data, and assemblage structure using Hellinger-transformed abundance data [42]. Hellinger transformation accounts for situations where sites that share no species may be geographically closer than sites that do share species [41]. *Post hoc* trend surface analysis of assemblage composition found significant spatial autocorrelation in the abundance data (Figure S1), and so we controlled for the influence of trap location by holding the spatial coordinates of traps as a constant in the ordinations [43]. Significance was tested through permutation tests using 999 randomisations. Analysis of similarities (ANOSIM [44]) was used to test for significance differences among assemblages. All analyses were performed using program R 2.14.2 [45] using the vegan package [46].

## Results

We captured 3,634 individuals of 53 species and 12 genera from 4,200 trap hours (Table S1). There was no significant effect on species richness of proximity to the edge (*t* = -0.95, df = 15, *p* = 0.36) or whether traps were in the group of traps set on the first or second day (*t* = 1.59, df = 19.8, *p* = 0.13). Over half of the species (28 of 53) were sampled by all five baits. A further six species were common across four baits. Thirteen species were sampled by one bait only, all of which had ≤2 individuals. Excluding uncommon species with ≤5 individuals (leaving 35 out of 53 species), species sampled were remarkably consistent across all bait types, with baits sampling 94-100% of the common species.

**Figure 1:**
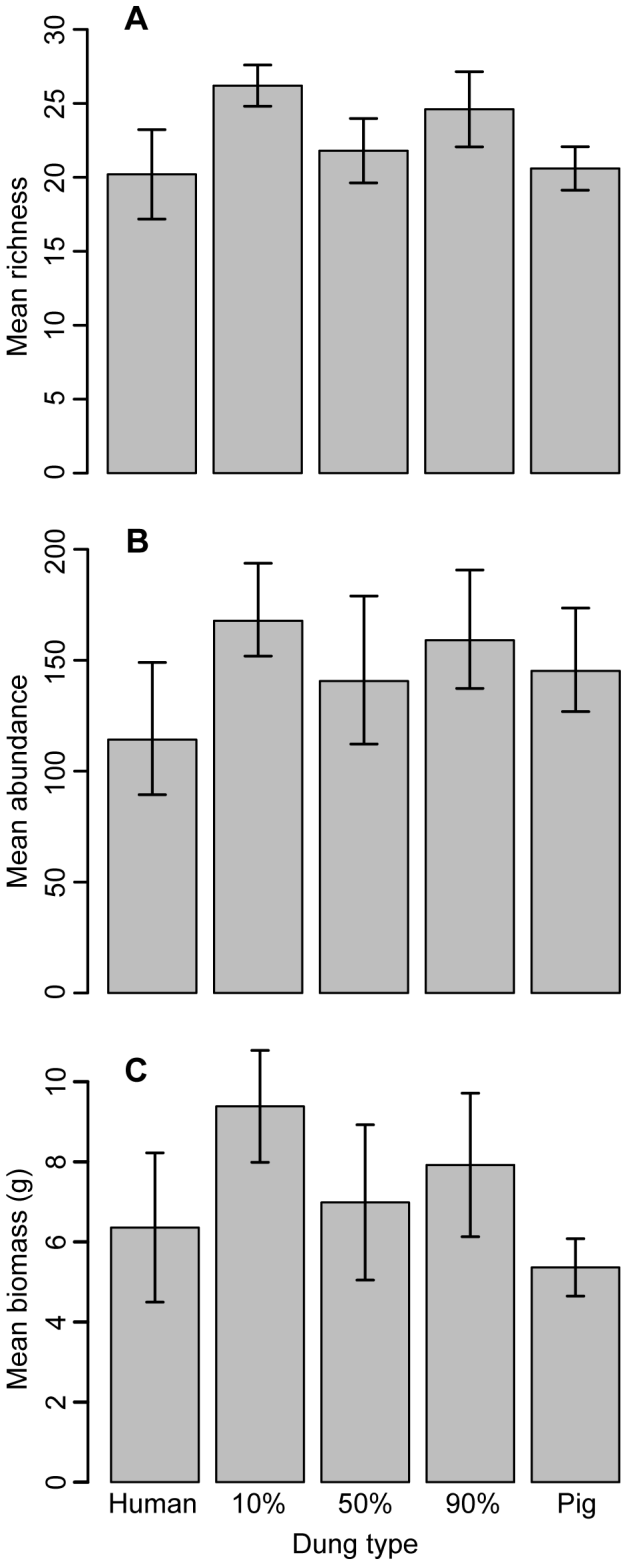
Mean number of species and individuals sampled by each bait type. Mean species richness (a), mean number of individuals (b) and mean biomass (c) of dung beetles sampled by each bait type. Pig-human bait mixes are referred to by the percentage contribution of pig dung to the overall mixture. Error bars represent the standard error.

**Figure 2:**
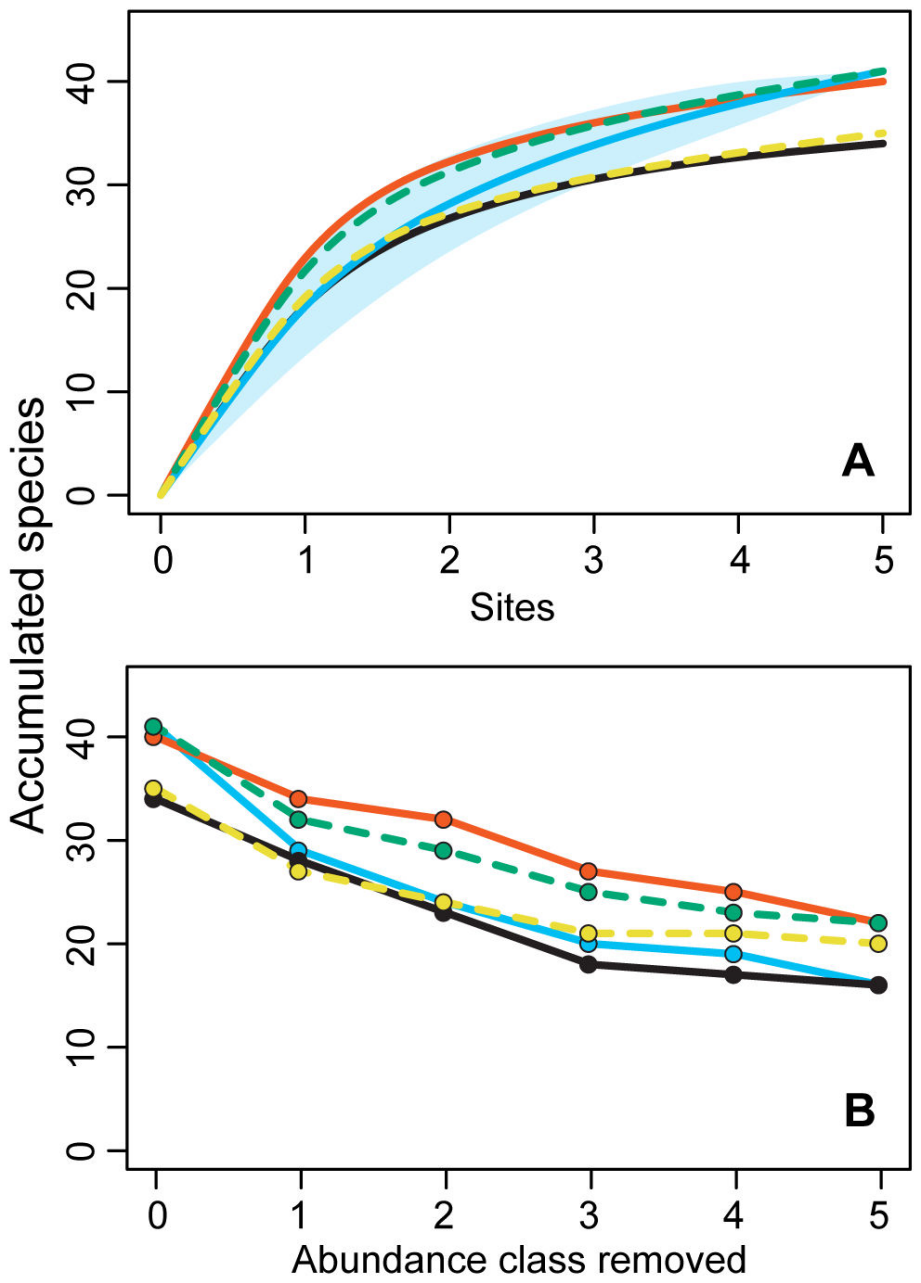
Species accumulation curves and the influence of occasional species for each bait type. Species accumulation curves (lines) for dung beetle communities (a), and numbers of species sampled by each of five bait types while sequentially removing singletons (abundance class = 1), doubletons (abundance class = 2) etc. (b). Colours represent dung type: human (blue), 10% pig (red), 50% pig (yellow dashed), 90% pig (green dashed), pig (black). The blue polygon represents the standard error for human dung.

Mean species richness, abundance and biomass was greater in the 10% and 90% pig mixes and lower in the two pure baits, as well as the 50% pig mix (Table 1, Figure 1), but there were no significant differences between baits. The site-based accumulation curves showed that the 10% and 90% mixes sampled at a faster rate than other dung mixes (Figure 2a, Figure S2), but that pure human dung captured a similar total number of species (75-77% of the total assemblage sampled; Table 1). The 50% mix and pure pig dung, however, both accumulated species at a slower rate and had lower total species richness (64-66% of the total assemblage). When rare species were removed, however, pure human dung sampled a relatively low number of species (Figure 2b). Almost 30% of species sampled by human dung were singletons and a further 12% doubletons (Table 1). If these species were removed from each bait then the 10% and 90% mixes have a much higher species richness (30 and 33 species respectively) compared to the other baits (23-24 species).

**Table 1:** Diversity indices for communities sampled by two pure dung baits and three mixes (given as the percentage of pig dung).

	**Human**	**10%**	**50%**	**90%**	**Pig**
**Mean richness (± se)**	20.2 (3.02)	26.2 (1.39)	21.8 (2.18)	24.6 (2.54)	20.6 (1.47)
**Total species**	41	40	35	41	34
**% of total species**	77.3%	75.5%	66%	77.3%	64.2%
**Mean individuals (± se)**	114.2 (29.8)	167.8 (20.9)	140.6 (33.4)	159 (26.7)	145.2 (23.3)
**Total individuals**	571	839	703	795	726
**Mean biomass/g (± se)**	6.36 (1.86)	9.38 (1.40)	6.99 (1.94)	7.92 (1.79)	5.36 (0.72)
**Singletons^a^**	12 (29.2%)	6 (15%)	8 (22.9%)	9 (22%)	6 (17.6%)
**Doubletons^a^**	5 (12.2%)	2 (5%)	3 (8.6%)	3 (7.3%)	5 (14.7%)
**Unique species^a^**	4 (9.8%)	4 (10%)	1 (2.9%)	3 (7.3%)	1 (2.9%)
**Evenness^b^**	2.74	2.92	2.63	2.92	2.31

^a^ Numbers in parentheses are absolute values as a proportion of the total number of species recorded by that bait type.

^b^ Measured through the Shannon Index.

**Figure 3:**
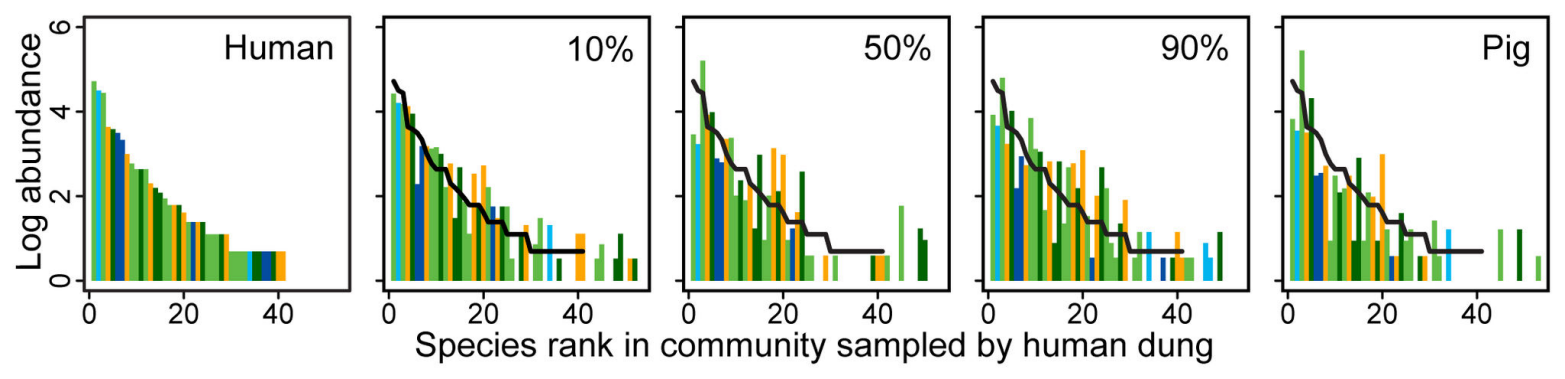
Rank abundance plots for species collected by each bait type. Rank abundance plots for species collected by different dung mixes. Bar colours indicate functional group: small rollers (green); large rollers (dark green); small tunnellers (blue); large tunnellers (dark blue); and dwellers (orange). On pig-based baits the rank abundance curve for pure human dung has been overlaid (black line) and abundance has been standardised to be equivalent to the total abundance of all species collected by pure human dung. Bars greater than the line indicate species with greater abundances than human dung, and bars below a lower abundance.

**Figure 4:**
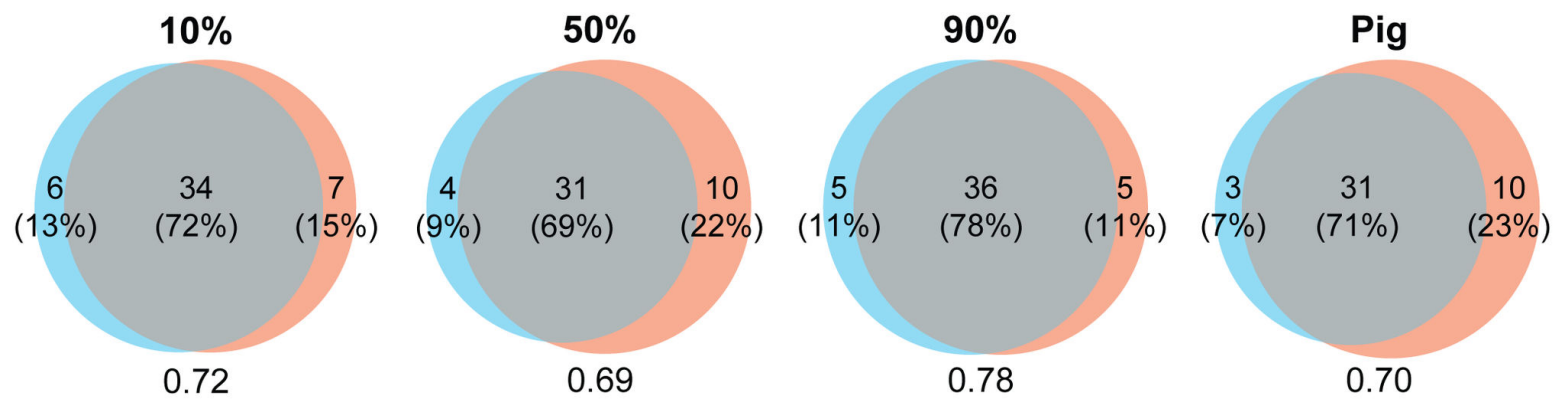
Proportion of shared species between the community sampled using human dung and by dung mixes. Proportional venn diagrams visualising the similarity between the community sampled using pure human dung and the communities sampled by dung mixes. Diagram components represent species unique to pure human dung (red), the number of species unique to the other bait type (blue), and species shared by both (overlap). Numbers below plots indicate similarity values (1-Jaccard) with human dung-baited traps.

The standardised rank abundance plots showed that common species were sampled by all baits in similar numbers (Figure 3). The identities of uncommon species, however, were variable between baits. 10% and 90% pig baits showed the most similar abundance patterns to human dung. This was reflected in the number of species shared with the human dung assemblage (Figure 4). All baits shared 69-78% of species with human-baited traps, with the 90% pig mix sharing the highest number of species (36). Furthermore, within-bait dissimilarities were very similar to between-bait dissimilarities (Table 2), suggesting that turnover between traps of the same bait were equivalent to if the traps had different baits.

**Table 2:** Mean Jaccard dissimilarities between traps with different baits and between traps with the same bait (italics).

	**Human**	**10%**	**50%**	**90%**	**Pig**
**Human**	*0.67*				
**10%**	0.64	*0.59*			
**50%**	0.73	0.68	*0.68*		
**90%**	0.68	0.62	0.65	*0.64*	
**Pig**	0.70	0.65	0.60	0.59	*0.52*

All baits trapped a similar richness and abundance of functional groups (Table 3), with the exception of large, crepuscular tunnellers, which were present only in pure human dung and the 10% mix. This groups consists of a single genus, *Coprophanaeus*, which are preferentially necrophagous but can occasionally be attracted to carnivorous or omnivorous dung [47]. All three species captured from this genus occurred as singletons. The other inconsistency occurred in the diurnal small roller group that had extremely low abundance in the 50% mix. This was the result of a single species, *Canthon* *triangularis* , that was very abundant in all other baits but not in the 50% mix, potentially representing a sampling anomaly.

**Table 3:** The number of species and individuals captured by each bait type for genera assigned to functional groups based upon breeding strategy and diel activity.

**Breeding strategy**	**Diel activity**	**Human**	**10%**	**50%**	**90%**	**Pig**
*Species richness*						
Dweller	Diurnal	9	10	9	9	7
Large roller	Crepuscular	5	3	3	4	3
Large tunneller	Diurnal	3	4	3	3	3
Large tunneller	Crepuscular	2	2			
Large tunneller	Nocturnal	5	5	6	6	5
Small roller	Diurnal	2	2	1	4	2
Small tunneller	Diurnal	10	9	8	10	8
Small tunneller	Nocturnal	5	5	5	5	6
*Abundance*						
Dweller	Diurnal	81	119	164	129	107
Large roller	Crepuscular	64	54	43	38	30
Large tunneller	Diurnal	21	43	36	58	16
Large tunneller	Crepuscular	2	2			
Large tunneller	Nocturnal	53	88	97	108	122
Small roller	Diurnal	90	101	3	59	46
Small tunneller	Diurnal	33	65	55	108	27
Small tunneller	Nocturnal	227	268	278	250	369

Partial canonical ordination on assemblage composition (presence data) was insignificant (adjusted *R* ^2^ = 0.02; *p* = 0.21). Ordination on assemblage structure (abundance data) was significant (*p* < 0.005), although the ordination explained just 16% of the variation in assemblage composition. The first axis explained 54.8% of the explained variation and the second axis 28.9%. Analysis of similarities found no significant differences in assemblage composition between dung types, but there were slight differences in assemblage structure between human dung and 50% pig dung (R = 0.376; *p* = 0.016) and pure pig dung (R = 0.364; *p* = 0.017).

## Discussion

Although there were small differences in the number of species and individuals sampled by each bait differences were largely non-significant. Furthermore, the assemblages sampled by each bait were remarkably consistent: all pig-based baits shared a very high proportion of species with the human-baited traps (Figure 4); nearly all functional groups occurred in similar numbers in each bait (Table 3); and nearly all of the species with ≥5 individuals were sampled by all baits (Figure 3). Those species that were confined to one or two bait types all occurred at very low abundances. This supports other investigations that have found a high number of generalists in dung beetle assemblages [20,28]. The composition of the sampled assemblages also did not differ markedly between baits, although there were slight differences in assemblage structure, with the assemblages sampled by the 10% and 90% mixes statistically consistent with the assemblage sampled by pure human dung. These results confirm that studies sampling using a pig-human dung mix would be comparable to studies using solely human dung bait, with the advantage of greatly increasing sampling effort.

Human dung-baited pitfalls have been shown to outperform herbivorous dung and carrion and fruit for sampling dung beetle assemblages [24,28], however, it has never been tested against pig dung or against dung mixes. Although mean richness was lower, human-baited pitfalls did collect an equivalent total number of species to the human-pig mixes, and more than traps baited with pure pig dung. This was primarily due to the large number of singletons and doubletons collected by human dung and the relatively slow rate of species accumulation, suggesting that a large number of species are only occasionally attracted to human dung whereas they may be caught in higher numbers by pig mixes (see the bars in Figure 3 for bait mixes that extend above the standardised rank abundance curve for the human-baited traps). Many studies exclude species with low abundance as although they may represent species that are naturally rare within the environment, they may also represent transient, tourist species (eg [18,19,47]). However, excluding these uncommon species may have important consequences for the interpretation of ecological results [39]. Almost 30% of species sampled by human dung were singletons and a further 12% doubletons (Table 1). In fact, if singletons were removed, then human dung-baited pitfalls collected no more species than those baited by pig dung.

## Conclusion

The richness, individual abundance, composition and structure of assemblages sampled by pig-human dung mixes were comparable to those sampled using pure human dung. From the baits that we tested, the 10% mix sampled the greatest number of species and individuals, and was compositionally closest to that of human dung. However with only a small addition of pig dung (2 g in a 20 g bait) there will still be practical issues with the availability of human dung that limit the number of traps that can be set. We recommend from these results that a 90:10 pig to human ratio, or a close proportion, represents a suitable compromise between accurately sampling the dung beetle assemblage, comparability with existing studies and the availability of large quantities of dung to facilitate expansive trap networks.

Our study investigated just two bait types at one location in Amazonian primary forest, yet nonetheless represents important information on the ability of dung mixes to adequately sample dung beetle assemblages. Further effort is required to test different dung types in different habitats and different continents with different pools of suitable bait sources to ascertain whether any particular bait or bait mix can be used in all investigations at any location. For example, domesticated pigs are not universally available, or the habitat of interest may favour a different bait; grassland and pasture species may show preferences to cow dung over pig dung [17]. Dung beetles are a promising indicator taxa for ecological research [3,5] and there have been recent attempts to carry out meta-analyses on the vast quantities of data already collected [6]. Assessing the comparability of assemblage samples collected using different baits, as we have done here, represents an important step towards facilitating rigorous comparisons among studies that have investigated the same taxon but without a standardised sampling protocol.

## Supporting Information

Figure S1 **Bubble plots of trap locations indicating species richness, abundance and spatial autocorrelation.** Bubble plots of trap locations. Bubble size represents species richness (a) and number of individuals (b) for each site. Colours represent dung type: human (red), 10% pig (orange), 50% pig (yellow), 90% pig (green), pig (dark green). There was significant spatial autocorrelation in abundance (c) but not for richness. Black bubbles represent positive spatial autocorrelation and white bubbles negative spatial autocorrelation.
(TIF)

Figure S2 **Individual-based species accumulation curves for each bait type.** Individual-based species accumulation curves (lines) for dung beetle communities. Colours represent dung type: human (blue), 10% pig (red), 50% pig (yellow dashed), 90% pig (green dashed), pig (black). The blue polygon represents the standard error for human dung.
(TIF)

Table S1 **The number of individuals for each species captured by each bait type.** Breeding strategy and diel activity is taken from Feer and Pincebourde [40].
(DOCX)
