# Supplementary figures and images for: Optimising Bait for Pitfall Trapping of Amazonian Dung Beetles (Coleoptera: Scarabaeinae)
Source: PLoS One. 2013 Aug 30;8(8):e73147. doi: 10.1371/journal.pone.0073147 (PMC3758266; doi:10.1371/journal.pone.0073147)

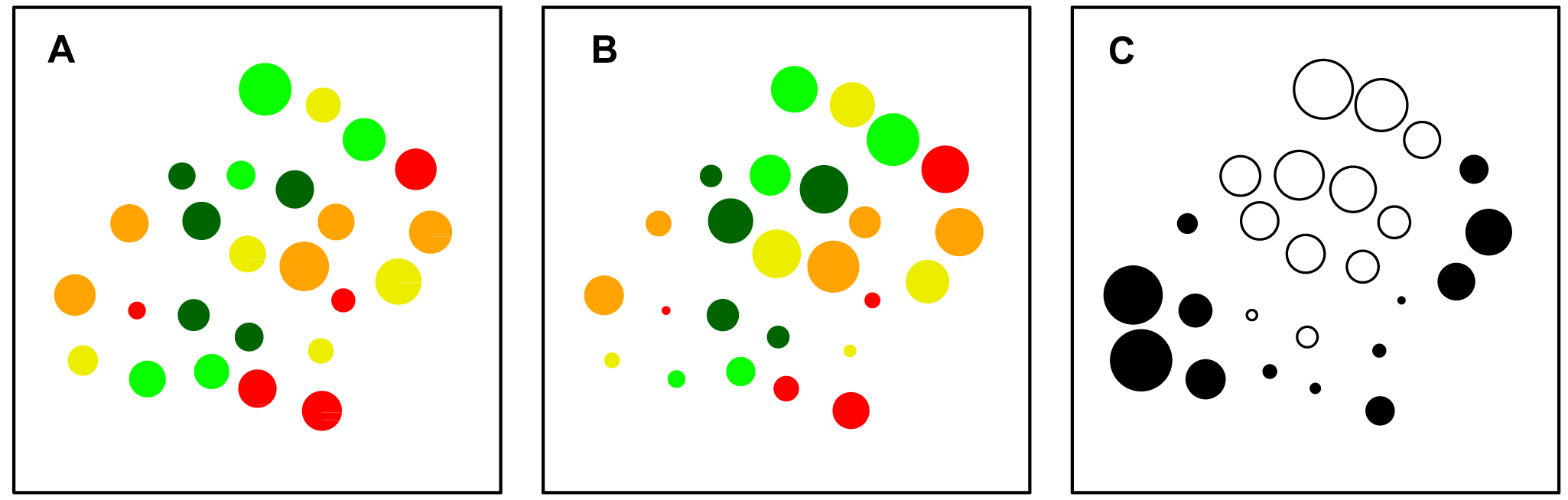

Supplement: Figure S1 — Bubble plots of trap locations indicating species richness, abundance and spatial autocorrelation. Bubble plots of trap locations. Bubble size represents species richness (a) and number of individuals (b) for each site. Colours represent dung type: human (red), 10% pig (orange), 50% pig (yellow), 90% pig (green), pig (dark green). There was significant spatial autocorrelation in abundance (c) but not for richness. Black bubbles represent positive spatial autocorrelation and white bubbles negative spatial autocorrelation. (TIF) [file pone.0073147.s001.tif]

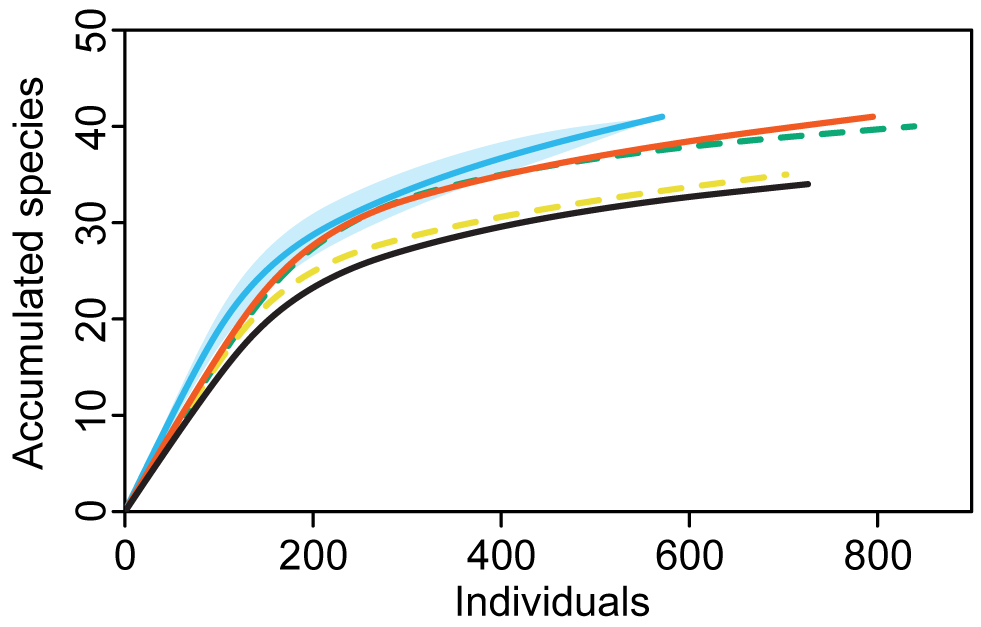

Supplement: Figure S2 — Individual-based species accumulation curves for each bait type. Individual-based species accumulation curves (lines) for dung beetle communities. Colours represent dung type: human (blue), 10% pig (red), 50% pig (yellow dashed), 90% pig (green dashed), pig (black). The blue polygon represents the standard error for human dung. (TIF) [file pone.0073147.s002.tif]
