# Supplementary material for: Optimising Bait for Pitfall Trapping of Amazonian Dung Beetles (Coleoptera: Scarabaeinae)
Source: PLoS One. 2013 Aug 30;8(8):e73147. doi: 10.1371/journal.pone.0073147 (PMC3758266; doi:10.1371/journal.pone.0073147)
Supplement: Table S1 — The number of individuals for each species captured by each bait type. Breeding strategy and diel activity is taken from Feer and Pincebourde [40]. (DOCX) [file pone.0073147.s003.docx]

**Table S1**

| **Species** | **Human** | **10** | **50** | **90** | **Pig** | **Breeding strategy** | **Diel activity** |
| --- | --- | --- | --- | --- | --- | --- | --- |
| Ateuchus aff. connexus | 5 | 3 | 8 | 19 | 9 | Small tunneller | Diurnal |
| Ateuchus aff. murrayi | 2 | 7 | 1 | 11 | 2 | Small tunneller | Diurnal |
| Ateuchus irinus | 15 | 32 | 35 | 64 | 2 | Small tunneller | Diurnal |
| Ateuchus pauki | 1 |  |  |  |  | Small tunneller | Diurnal |
| Ateuchus sp. A |  |  | 1 | 1 |  | Small tunneller | Diurnal |
| Ateuchus sp. B |  |  |  | 1 |  | Small tunneller | Diurnal |
| Ateuchus sp. E | 1 | 2 | 1 | 1 | 4 | Small tunneller | Diurnal |
| Ateuchus sp. F |  | 1 |  |  |  | Small tunneller | Diurnal |
| Ateuchus sp. G | 1 | 5 |  | 3 | 1 | Small tunneller | Diurnal |
| Canthidium aff. gertsaeckeri | 2 | 1 | 1 | 2 | 3 | Small tunneller | Diurnal |
| Canthidium aff. lentum | 3 | 12 | 2 | 5 | 3 | Small tunneller | Diurnal |
| Canthidium sp. B |  | 2 | 6 |  | 3 | Small tunneller | Diurnal |
| Canthidium sp. D | 1 |  |  |  |  | Small tunneller | Diurnal |
| Canthidium sp. H | 2 |  |  | 1 |  | Small tunneller | Diurnal |
| Canthon bicolor | 1 | 4 |  | 3 | 3 | Small roller | Diurnal |
| Canthon quadriguttatus |  |  |  | 2 |  | Small roller | Diurnal |
| Canthon subhyalinus |  |  |  | 1 |  | Small roller | Diurnal |
| Canthon triangularis | 89 | 97 | 3 | 53 | 43 | Small roller | Diurnal |
| Coprophanaeus dardanus |  | 1 |  |  |  | Large tunneller | Crepuscular |
| Coprophanaeus jasius | 1 |  |  |  |  | Large tunneller | Crepuscular |
| Coprophanaeus lancifer | 1 | 1 |  |  |  | Large tunneller | Crepuscular |
| Deltochilum aff. peruanum | 27 | 34 | 19 | 25 | 15 | Large roller | Crepuscular |
| Deltochilum aff. submetallicum | 32 | 13 | 21 | 11 | 14 | Large roller | Crepuscular |
| Deltochilum carinatus | 3 | 7 | 3 | 1 | 1 | Large roller | Crepuscular |
| Deltochilum icarus | 1 |  |  | 1 |  | Large roller | Crepuscular |
| Deltochilum orbiculare | 1 |  |  |  |  | Large roller | Crepuscular |
| Dichotomius apicalis | 2 | 3 |  | 4 | 1 | Large tunneller | Nocturnal |
| Dichotomius boreus | 1 |  | 1 | 1 |  | Large tunneller | Nocturnal |
| Dichotomius latilobatus |  | 3 | 3 | 3 | 3 | Large tunneller | Nocturnal |
| Dichotomius lucasi | 35 | 75 | 65 | 76 | 94 | Large tunneller | Nocturnal |
| Dichotomius mamillatus |  |  | 2 |  |  | Large tunneller | Nocturnal |
| Dichotomius subaeneus | 7 | 2 | 23 | 22 | 22 | Large tunneller | Nocturnal |
| Dichotomius worontzowi | 8 | 5 | 3 | 2 | 2 | Large tunneller | Nocturnal |
| Eurysternus atroserucus | 19 | 34 | 34 | 2 | 18 | Dweller | Diurnal |
| Eurysternus balachowskyi | 1 | 3 | 1 | 3 |  | Dweller | Diurnal |
| Eurysternus caribaeus | 37 | 9 | 61 | 34 | 41 | Dweller | Diurnal |
| Eurysternus cayennensis |  | 1 |  |  |  | Dweller | Diurnal |
| Eurysternus foedus | 5 | 17 | 27 | 21 | 8 | Dweller | Diurnal |
| Eurysternus hamaticollis | 1 | 3 | 1 | 1 |  | Dweller | Diurnal |
| Eurysternus hypocryta | 9 | 22 | 11 | 22 | 14 | Dweller | Diurnal |
| Eurysternus strigulatus | 4 | 21 | 23 | 29 | 24 | Dweller | Diurnal |
| Eurysternus vastorium | 2 | 4 | 1 | 8 | 1 | Dweller | Diurnal |
| Eurysternus ventricosus | 3 | 5 | 5 | 9 | 1 | Dweller | Diurnal |
| Onthophagus aff. bidentatus | 13 | 33 | 8 | 3 | 14 | Small tunneller | Nocturnal |
| Onthophagus aff. clypeatus | 111 | 121 | 38 | 69 | 57 | Small tunneller | Nocturnal |
| Onthophagus aff. haemathopus | 6 | 8 | 2 | 4 | 2 | Small tunneller | Nocturnal |
| Oxysternon durantoni | 3 | 7 | 15 | 19 | 5 | Large tunneller | Diurnal |
| Oxysternon festivum | 13 | 28 | 12 | 28 | 9 | Large tunneller | Diurnal |
| Phanaeus chalcomelas | 5 | 7 | 9 | 11 | 2 | Large tunneller | Diurnal |
| Sulcophanaeus faunus |  | 1 |  |  |  | Large tunneller | Diurnal |
| Uroxys sp. A | 84 | 94 | 223 | 168 | 294 | Small tunneller | Nocturnal |
| Uroxys sp. B |  |  |  |  | 1 | Small tunneller | Nocturnal |
| Uroxys sp. C | 13 | 12 | 7 | 6 | 1 | Small tunneller | Nocturnal |
